# FOXA1 Suppresses the Growth, Migration, and Invasion of Nasopharyngeal Carcinoma Cells through Repressing miR-100-5p and miR-125b-5p

**DOI:** 10.7150/jca.40709

**Published:** 2020-02-10

**Authors:** Qian Peng, Liyang Zhang, Junjun Li, Wei Wang, Jing Cai, Yuanyuan Ban, Ying Zhou, Meng Hu, Yan Mei, Zhaoyang Zeng, Xiaoling Li, Wei Xiong, Guiyuan Li, Yixin Tan, Bo Xiang, Mei Yi

**Affiliations:** 1 Hunan Provincial Cancer Hospital and Cancer Hospital Affiliated to Xiangya Medical School, Central South University, Changsha 410013, Hunan, China; 2 The Key Laboratory of Carcinogenesis and Cancer Invasion of the Chinese Ministry of Education, Cancer Research Institute and School of Basic Medical Sciences, Central South University, Changsha, 410078, Hunan, China; 3 The Key Laboratory of Carcinogenesis of the National Health Commission, Xiangya Hospital, Central South University, Changsha 410078, Hunan, China; 4 Hunan Key Laboratory of Nonresolving Inflammation and Cancer, Disease Genome Research Center, The Third Xiangya Hospital, Central South University, Changsha, Hunan, China; 5 Department of Neurosurgery, Xiangya Hospital, Central South University, Changsha, 410008, Hunan, China.; 6 Department of Pathology, Affiliated Hospital of Jining Medical University, Jining, 272029, Shandong, China; 7 Department of Dermatology, Second Xiangya Hospital, The Central South University, Hunan Key Laboratory of Medical Epigenetics, Changsha, 410011, Hunan, China; 8 Department of Dermatology, Xiangya Hospital of Central South University, Changsha, 410008, China

**Keywords:** nasopharyngeal carcinoma, FOXA1, microRNA, miR-100, miR-125b

## Abstract

**Background**: Nasopharyngeal carcinoma (NPC) is a unique subtype of head and neck cancer, within highest incidence in South China and southeastern Asia but rare in other regions worldwide. FOXA1 is a pioneer factor implicated in various human malignancies. Downregulation of FOXA1 promotes NPC cells proliferation, invasiveness in vitro and tumorigenicity in vivo. However, it is remain elusive to determine whether microRNAs (miRNAs) regulated by FOXA1 contribute to NPC progression.

**Methods**: In this study, differentially expressed miRNAs and mRNAs induced by FOXA1 expression were determined by microarray. Integrative miRNA-mRNA regulatory networks mediated by FOXA1 in NPC were established. The expressions of differentially expressed miRNAs in NPC cells were measured by quantitative reverse-transcription PCR. Cell viability was determined by CCK-8 assays. Cell migration and invasiveness were measured by Transwell assays. The correlation between miRNAs and its target mRNAs was analyzed.

**Results**: FOXA1 suppressed the expression of miR-100-5p and miR-125b-5p in NPC cells. Silencing either miR-100-5p or miR-125b-5p inhibited the malignant behaviors of NPC cells, whereas re-expression of miR-100-5p or miR-125b-5p restored the malignancy of NPC cells repressed by FOXA1. Mechanistically, miR-100-5p or miR-125b-5p suppressed RASGRP3 or FOXN3 expression respectively via direct binding to its 3'-UTR. Furthermore, we demonstrated that FOXA1 induced RASGRP3 or FOXN3 expression via inhibiting miR-100-5p or miR-125b-5p. Upregulation of RASGRP3 or FOXN3 contributed to inhibition of NPC by FOXA1. We also demonstrated that the mRNA levels of RASGRP3 and FOXN3 are positively correlated with FOXA1.

**Conclusion**: Our study provided evidence the first time that FOXA1 suppresses NPC cells via downregulation of miR-100-5p or miR-125b-5p.

## Introduction

Nasopharyngeal carcinoma (NPC) is a unique subtype of head and neck cancer which has the highest incidence in south China and eastern south Asia but rare in other regions worldwide 1, 2. Several etiological factors are implicated in development of NPC, including latent infection of Epsterin-Barr virus (EBV), exposure of chemical carcinogens and genetic susceptibility 3, 4. There are some common symptoms of NPC, including nasal obstruction, bloody mucus, ear stuffy, hearing loss, diplopia, and headache. Due to NPC's high sensitivity to radiotherapy, radiotherapy is acknowledged as the preferred effective treatment for NPC 5. Furthermore, radiotherapy cooperated with chemotherapy has provided much better results for NPC according to recent scientific research and clinical trials 6-8.

Forkhead box A1 (FOXA1), also known as hepatocyte nuclear factor 3-alpha (HNF-3A) or transcription factor 3a (TCF3A), is a member of forkhead family that encode DNA-binding proteins. FOXA1 is classified as a pioneer factor that are a kind of transcription factors directly binding condensed chromatin. FOXA1 binds to compacted chromatin regions and recruits chromatin remodeling complex to establish an opened chromatin architecture, facilitating other lineage specific transcription factors binding 9, 10. FOXA1 has been shown to be implicated in diverse cancer development, such as breast cancer, prostate cancer and bladder cancer. Recurrent mutations of FOXA1 have been frequently detected in prostate cancer 11, 12. Our previous studies reported that FOXA1 in highly expressed in normal nasopharyngeal epithelium but decreased in NPC samples 13, 14. Restoration of FOXA1 in NPC cells inhibits cell proliferation, invasiveness in vitro and tumorigenicity in vivo. In the molecular level, FOXA1 reprograms TGF-β-stimulated transcriptional program to favor the growth inhibitory effect of TGF-β on NPC cells growth 15. So far, no publication reported whether FOXA1 modulate microRNAs (miRNAs) expression to inhibit NPC development.

MicroRNA is the small non-coding RNA that contains approximately twenty-two nucleotides. miRNAs regulate the gene expression through binding to the mRNA and facilitating its degradation or preventing its translation efficiency 16. Massive studies revealed that miRNAs are dysregulated in diverse human cancers and implicated in cancer development and progression 17.

In this study, we demonstrated for the first time that FOXA1 suppressed the malignant behaviors of NPC cells through repressing two oncogenic miRNAs, miR-100-5p and miR-125b-5p.

## Method and Materials

### Cell culture

Human nasopharyngeal carcinoma cell lines HK1 and CNE1 were cultured in RPMI-1640 medium containing 10% fetal bovine serum (FBS) and penicillin/streptomycin (Gibco, Grand Island, NY). Cells were incubated at 37 °C with 5% CO_2_ and 95% air. Human embryonal kidney (HEK) 293T cells were cultured in Dulbecco's modified Eagle's medium (DMEM) containing 10% FBS and incubated at 37 °C with 5% CO_2_ and 95% air.

### miRNA mimics, inhibitors, siRNA and gene transfection

The miRNA mimics of miR-100-5p and miR-125b-5p were purchased from RiboBio (RiboBio Co.Ltd, Guangzhou, China). Lentivirus containing inhibitors for miR-100-5p and miR-125b-5p were purchased from Shanghai GenePharma Co. Ltd (Shanghai, China). The siRNAs targeting RASGRP3, FOXN3, and nonspecific siRNAs (scrambled sequences) were purchased from Shanghai GenePharma Co. Ltd (Shanghai, China). The siRNA sequences were listed as follows: siRASGRP3: 5'-GCAAUUACCGCAAGGCCUUUG-3' and 5'-CAAAGGCCUUGCGGUAAUUGC-3'; siFOXN3: 5'-GGAGUCAGAGUAUUGGGAATT-3' and 5'-UUCCCAAUACUCUGACUCC-3'; Stealth siRNA Negative Control: 5'-UUCUCCGAACGUGUCACGUTT-3' and 5'-ACGUGACACGUUCGGAGAATT-3'.

Transfection of miRNA mimics or siRNAs were performed by using Lipofectamine^®^ RNAiMAX Reagent (Invitrogen, Carlsbad, CA, USA) according to the manufacture's protocol.

### RNA extraction, reverse transcription and real-time quantitative PCR assay

Total cellular RNAs were extracted by using TRIzol Reagent (Invitrogen, Carlsbad, CA, USA) according to the manufacturer's instructions. Then the total RNA was treated with DNase Ⅰ (TaKaRa Biomedicals, Tokyo, Japan) to remove residual contamination of genomic DNA, and was purified in accordance with the instruction. For revert transcription, 3 μg of total RNA was reverse-transcribed into cDNA by using RevertAid First Strand cDNA Synthesis Kit (Fermentas, Thermo Fisher Scientific, Inc., Waltham, MA) in accordance with the manufacturer's protocol. Poly(A) tailing, reverse transcription and RT-PCR quantification of miRNAs were performed by using miDETECT A TrackTM miRNA qRT-PCR Starter Kit (RiboBio, Guangzhou, China) according to the manufacturer's protocol. Primer sets for miRNAs qPCR quantification were purchased from RiboBio Co. (Guangzhou, China). Real-time quantitative PCR reactions with 2× SYBR Green qPCR Master Mix (Selleckchem, Houston, TX, USA) were carried out by CFX96 Two-step Real-Time PCR Detection System (Bio-Rad, Hercules, CA). Relative transcripts expression was calculated with the 2^-ΔΔCt^ method.

### Target genes prediction and KEGG pathway analysis

TargetScan 7.2 (http://www.targetscan.org/vert_72/), a software and database specializing in the analysis of target genes of mammalian miRNA, was used to predict target genes of miR-100-5p and miR-125b-5p. The other two commonly used predicting websites, miRTarBase (http://mirtarbase.mbc.nctu.edu.tw/php/index.php) and miRDB (http://www.mirdb.org/), were also made use of for prediction. Venny 2.1.0 (http://bioinfogp.cnb.csic.es/tools/venny/) was used to overlap predicted target genes by TargetScan 7.2 with miRNA microarrays. KEGG pathway analysis was performed by DAVID 6.7 (https://david-d.ncifcrf.gov/tools.jsp).

### Cell Counting Kit-8 (CCK-8) assay

NPC HK1 cells were seeded at a density of 1×10^3^ cells per well in 96-well plates and were cultured at 37 °C for various times. Relative cell growth was assessed by CCK-8 assay (Bimake, Shanghai, China) according to manufacturer's instruction.

### Protein extraction and Western blotting

Total cellular proteins were extracted by using the RIPA lysis buffer (Solarbio Bioscience &Technology Co., Ltd., Shanghai, China) containing Protease Inhibitor Cocktail (Bimake, Shanghai, China). Cell lysates were centrifuged and collected proteins were immediately used or temporarily stored at -80 °C.

Cellular proteins concentration was assessed by using BCA Protein Assay Kit (Beyotime, China). Equal amounts of proteins were separated by SDS-PAGE gel electrophoresis and transferred onto PVDF membranes (Millipore, Billerica, MA). The membranes were blocked with 5% non-fat milk dissolved in TBST at room temperature for 1 hour, and immunoblotted with indicated antibodies below at 4 °C overnight: polyclonal anti-RASGRP3 (Abclonal; Cat No. A7791), polyclonal anti-FOXN3 (Absin; Cat No. abs103848) and anti-GAPDH (Abclonal; Cat No. AC033). After incubation with peroxidase-conjugated secondary antibodies, immunoblotting was visualized by using ECL-plus (Amersham Biosciences). Luminescent signal was detected by MiniChemi^TM^ Chemiluminescence imager (Beijing Sage Creation Science Co.).

### miRNA and mRNA microarrays

TRIzol reagent (Invitrogen) was employed to extract cellular RNAs. Preparation of cDNA and cRNA was performed in accordance with guidance of the Affymetrix GeneChip Expression Analysis Manual (Affymetrix, Santa Clara, CA). FOXA1 induced global changes on miRNA or mRNA expressions were compared by using Affymetrix miRNA 4.0 Array and Affymetrix Human Genome U133 Plus 2.0 microarrays, respectively. Differentially expressed miRNAs or mRNAs between different cell populations were identified by a twofold cutoff.

### Cell migration and invasion assays

Transwell assays by using 8-μm-pore Transwell inserts (Corning-Costar, Cambridge, MA, USA) pre-coated with or without 15 μL of Matrigel (BD Biosciences, Bedford, MA, USA) were employed to assess tumor cell invasion or migration, respectively. Briefly, single cell suspensions (1×10^5^ cells) in serum free RMPI-1640 medium (Gibco) were into the upper insert, which were loaded into the lower chamber with serum containing medium. Transwell inserts were incubated for 6-24 h at 37 °C, allowing cells migration or invasion across the inserts membrane. The migrated or invaded cells were stained by crystal violet dye and counted under microscope. Cell numbers from five random fields were counted.

### In vivo tumorigenesis

Tumor cell suspensions (1×10^6^ cells/0.2 mL) were subcutaneously inoculated into 6-week-old male BALB/c nude mice (Shanghai SLAC Laboratory Animal Co. Ltd., Shanghai, China), Animal experiments were performed in accordance with guidance approved by the Institutional Animal Care and Use Committee (IACUC) of Central South University.

### Dual-luciferase reporter assays

Potential binding sites of target genes for their corresponding miRNAs were predicted by TargetScan 7.2. Then the wild type and mutant type of the potential binding sites at 3' UTR of target genes were designed and synthetized by Sangon Biotech (Shanghai) Co., Ltd. The fragments were combined with dual-luciferase reporter vectors by double enzyme digestion. Transfection and fluorescence detection were performed by Dual-Luciferase ® Reporter Assay System (Promega Corporation, an affiliate of Promega (Beijing) Biotech Co., Ltd.) according to the manufacturer's instructions.

### Statistical analysis

The Student's t test was employed to compare the quantitative variables differences between groups. The SPSS 13.0 software package (SPSS, Chicago, IL, USA) was used for statistical analysis. A value of P<0.05 was considered statistically significant.

## Results

### Interactive network of miRNAs-mRNA regulated by FOXA1 expression in NPC cells

**Figure 1:**
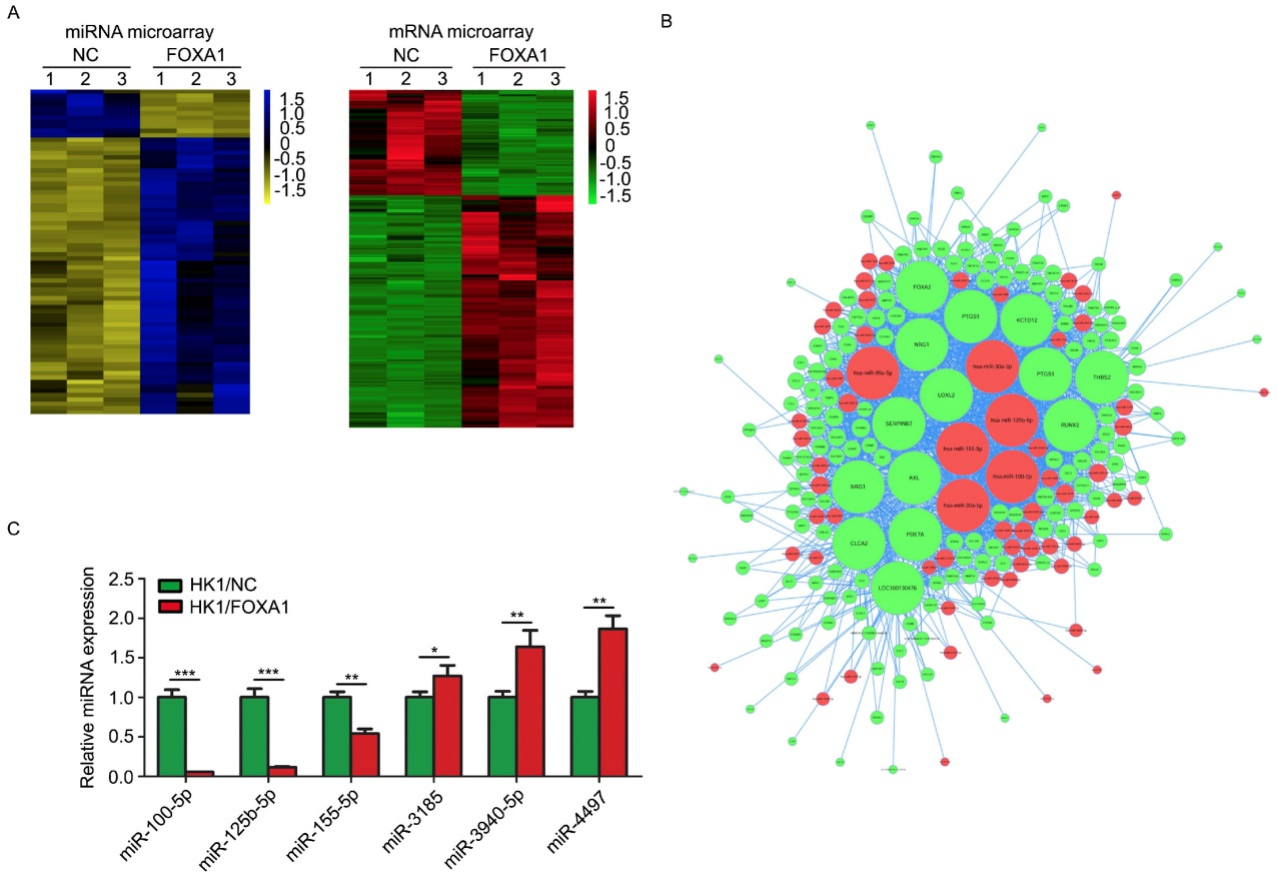
** miR-100-5p and miR-125b-5p were down-regulated in HK1 cells overexpressing FOXA1.** A, representative heatmaps showed differentially expressed miRNAs or mRNAs in control or HK1/FOXA1 cells. B. intergrative miRNA-mRNA network regulated by FOXA1 in HK1 cells. C, RT-PCR assay showed the validation of differentially expressed miRNAs in control or HK1/FOXA1 cells. *P < 0.05, **P < 0.01, ***P < 0.001.

FOXA1 stable expressing HK1 and CNE1 cells were established previously 15. FOXA1 protein levels in HK1 and CNE1 were measured by western blot assays (Figure S1). We used HK1/NC and HK1/FOXA1 cells to compare the molecular alterations induced by FOXA1. Based on stringent filtering approach (adjusted *P* value < 0.05, fold change > 2 or < 1/2), we identified 63 upregulated and 11 downregulated miRNAs and 211 upregulated and 87 downregulated mRNAs upon restoration of FOXA1 in NPC HK1 cells. Based on some differentially expressed mRNAs and all differentially expressed miRNAs we found that restoration of FOXA1 led to upregulation of 63 miRNAs, whereas downregulation of 11 miRNAs in HK1 cells (Figure 1A). The potential target genes of FOXA1 regulated miRNAs were determined by TargetScan software. Given changes in differentially expressed mRNAs may be induced by the regulation of miRNAs, and the integrative miRNA-mRNA regulatory network was constructed by Cytoscape based on the overlapping the predicted targets and differentially expressed mRNAs (Figure 1B). Three miRNAs with the greatest difference among the down-regulated (hsa-miR-100-5p, hsa-miR-125b-5p, hsa-miR-155-5p) and three miRNAs with the greatest difference among the up-regulated (hsa-miR-3185, hsa-miR-3940-5p, hsa-miR-4497) were chosen for further RT-PCR validation. As shown in Figure 1C, two miRNAs, miR-100-5p and miR-125b-5p, showed the dramatic reduction upon FOXA1 expression in HK1 (Figure 1C), suggesting strong functional relevance. RT-PCR assays demonstrated that forced expression of FOXA1 also led to remarkable reduction of miR-100-5p and miR-125b-5p expression levels in CNE1 cell (Figure S2). Therefore, the two miRNAs were selected for further study.

### Inhibition of miR-100-5p and miR-125b-5p suppressed malignancy of NPC cells in vitro

**Figure 2:**
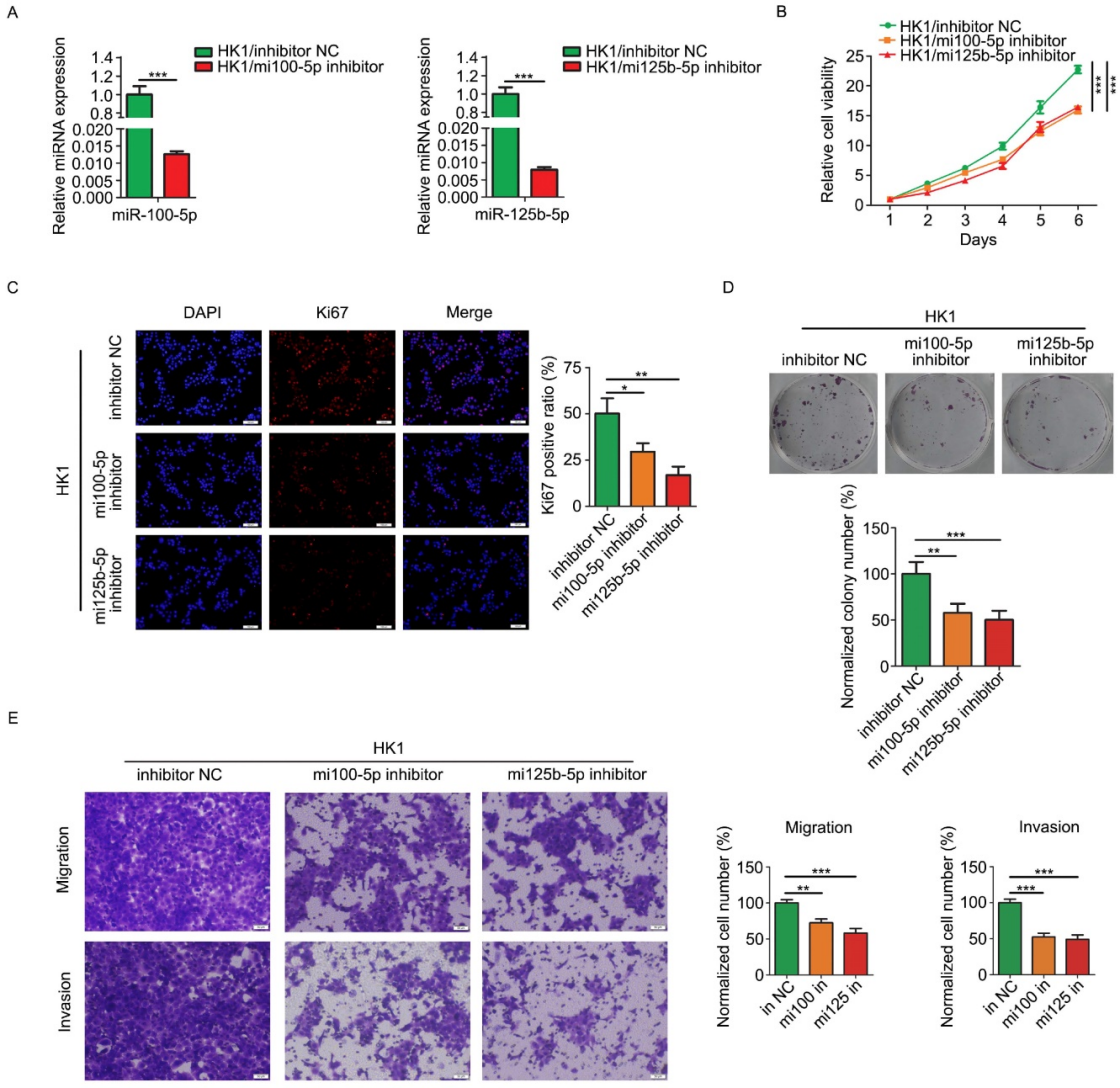
** Loss of miR-100-5p or miR-125b-5p inhibited cell proliferation, migration and invasion of NPC HK1 cells in vitro.** A, miR-100-5p and miR-125b-5p levels were determined by RT-PCR. B, CCK-8 assay showed that loss of miR-100-5p or miR-125b-5p suppressed cell viability in HK1 cells. C, Ki67 immunofluorescence assay showed that the depletion of miR-100-5p or miR-125b-5p reduced proliferating cell numbers in HK1 cells. D. colony formation assays showed that inhibition of miR-100-5p or miR-125b-5p suppressed tumor cell survival in HK1 cells. E, Transwell assays showed loss of miR-100-5p or miR-125b-5p impaired cell migration and invasiveness. *P < 0.05, **P < 0.01, ***P < 0.001.

Loss of function strategy was employed to determine the biological function of miR-100-5p and miR-125b-5p in NPC cells. HK1 cells were stably infected with lentivirus expressing inhibitors for either miR-100-5p or miR-125b-5p (referred to simply as mi100-5p inhibitor and mi125b-5p inhibitor). RT-PCR assay revealed that the expression of miR-100-5p or miR-125b-5p was effectively reduced by inhibitors (Figure 2A). CCK-8 assays revealed that silencing either miR-100-5p or miR-125b-5p significantly inhibited the growth of HK1 cells (Figure 2B). In addition, depletion of either miR-100-5p or miR-125b-5p in HK1 cells led to significant reduction of the frequency of Ki67 positive cells (Figure 2C). Furthermore, colony formation assays revealed that there are less colonies formed in miR-100-5p or miR-125b-5p silenced cells than control HK1 cells, suggesting reduced colony formation ability upon depletion either miR-100-5p or miR-125b-5p (Figure 2D). We further measured the effect of miR-100-5p or miR-125b-5p on cell migration and invasiveness by transwell chamber. As shown in Figure 2E, the number of cells passing through the transwell membrane in either miR-100-5p or miR-125b-5p silenced cells was much lower than that in the control HK1 cells, suggesting that loss of expression of either miR-100-5p or miR-125b-5p impair migratory and invasive ability of HK1 cells.

### Inhibition of miR-100-5p and miR-125b-5p attenuated the tumorigenicity of NPC cells in vivo

**Figure 3:**
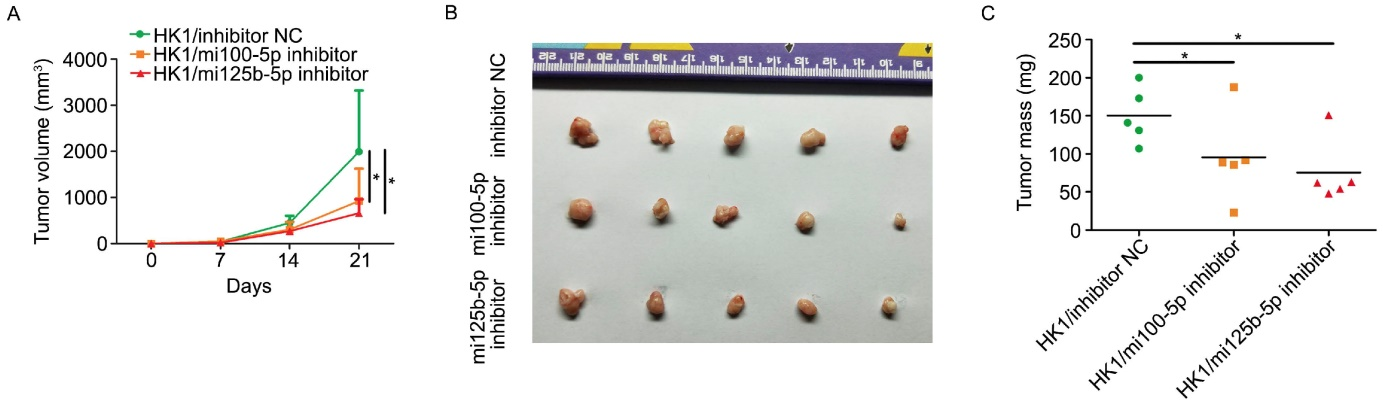
** repression of either miR-100-5p or miR-125b-5p suppressed tumorigenicy of NPC in vivo.** A, growth curve of subcutaneous xenograft tumor showed that loss of either miR-100-5p or miR-125b-5p suppressed tumorigenicity in HK1 cells. B, photograph showed xenograft tumors from control cells or HK1 cells lacking either miR-100-5p or miR-125b-5p. C, measurement of xenograft tumor mass. *P < 0.05.

The effect of silencing either miR-100-5p or miR-125b-5p on tumorigenicity of HK1 cells was determined by subcutaneous xenograft tumor assay. As shown in Figure 3A, xenograft tumors formed by either miR-100-5p or miR-125b-5p silenced cells grow more slowly than that of formed by control HK1 cells. At the endpoint of experiment, nude mice were sacrificed and subcutaneous xenograft tumors were removed. As shown in Figure 3B and 3C, the tumors formed by either miR-100-5p or miR-125b-5p silenced cells were smaller than that from control cells. Taken together, our data indicated that miR-100-5p and miR-125b-5p exert oncogenic activity in NPC development.

### FOXA1 suppressed the malignancy of NPC cells via inhibition of miR-100-5p and miR-125b-5p

**Figure 4:**
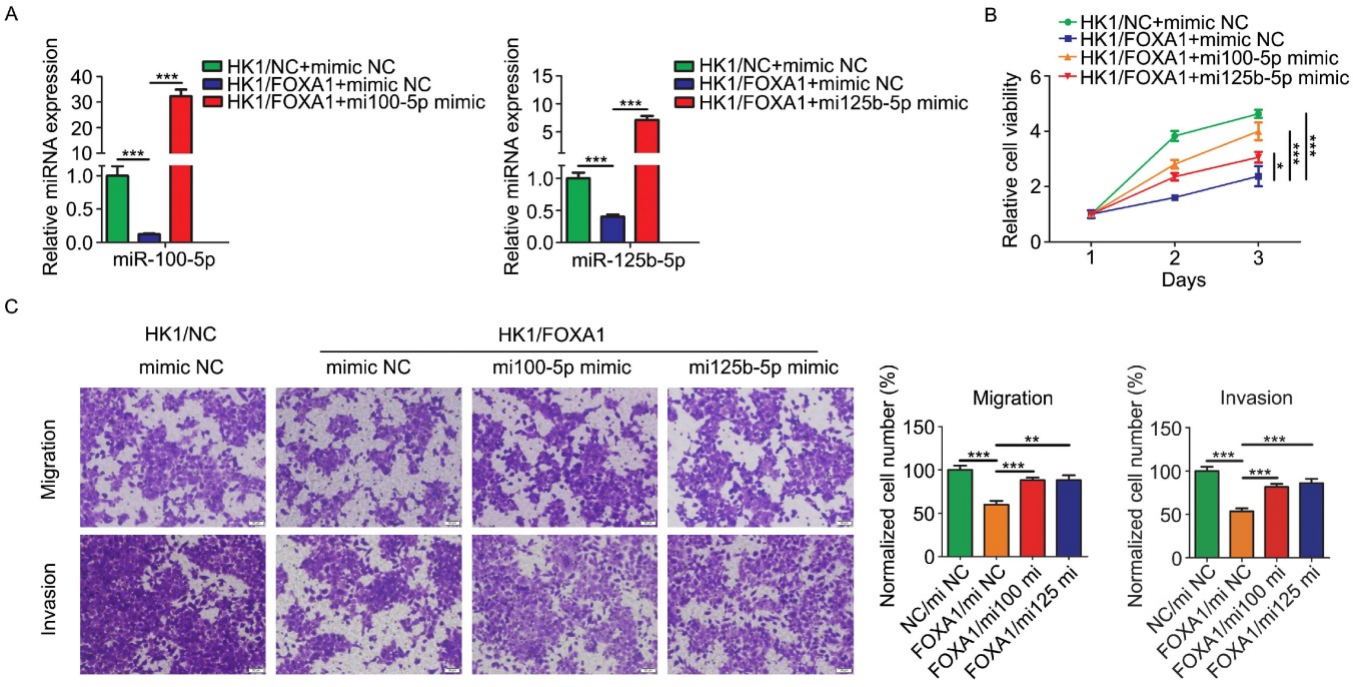
** Re-expression of miR-100-5p or miR-125b-5p restored HK1 cells proliferation, migration and invasion suppressed by FOXA1.** A, miR-100-5p and miR-125b-5p miRNA levels in HK1/FOXA1 cells transfected instantaneously with the mimics of miR-100-5p or miR-125b-5p were determined by RT-PCR. B, CCK-8 assay showed that transfection of either miR-100-5p or miR-125b-5p mimics led to recovery of cell viability in HK1/FOXA1 cells. C, Transwell assays showed either miR-100-5p or miR-125b-5p mimics enhanced migration or invasiveness in HK1/FOXA1 cells. *P < 0.05, **P < 0.01, ***P < 0.001.

In order to determine the biological function relevance between FOXA1 and miR-100-5p or miR-125b-5p, we asked whether restoration of miR-100-5p and miR-125b-5p expression by transfection of miRNA mimics (referred to simply as mi100-5p mimic and mi125b-5p mimic) could rescue tumor phenotype changes mediated by FOXA1 in HK1 cells. Real-time qPCR assay showed transfection of mimics effectively restored the expression level of either miR-100-5p or miR-125b-5p (Figure 4A & B). CCK-8 assay showed that FOXA1 suppressed the growth of HK1 cells, whereas transfection of either miR-100-5p or miR-125b-5p rescued tumor cell growth in HK1/FOXA1 cells (Figure 4C), indicating that downregulation of miR-100-5p and miR-125b-5p contribute to cell growth inhibition regulated by FOXA1. Then we tested the effects of miR-100-5p or miR-125b-5p on cells migration and invasiveness in HK1/FOXA1 cells. As shown in Figure 4D, expression of FOXA1 inhibited the migratory and invasive ability in HK1 cells, whereas restoration of either miR-100-5p or miR-125b-5p led to elevation of both migration and invasion in HK1/FOXA1 cells. Thus, our data suggested that FOXA1 exerts inhibitory effects on malignant behaviors at least partially through repression of miR-100-5p and miR-125b-5p.

### FOXA1 upregulates the expression of RASGRP3 or FOXN3 via inhibiting miR-100-5p and miR-125b-5p

**Figure 5:**
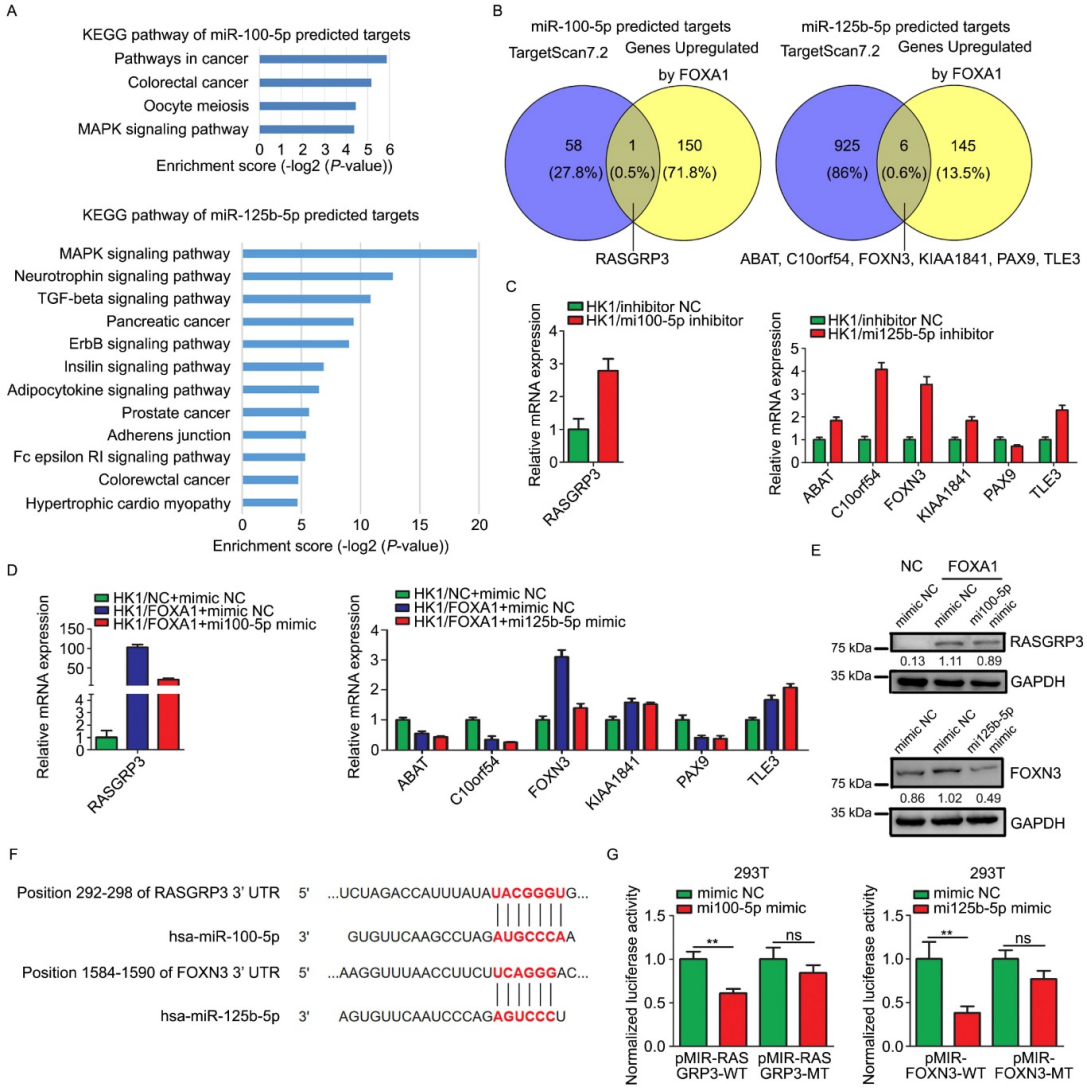
** miR-100-5p and miR-125b-5p targeted to RASGRP3 or FOXN3.** A, Enrichment KEGG pathways of miR-100-5p or miR-125b-5p target genes. Potential target genes of miRNAs were predicted by TargetScan 7.2. B, Venn diagram showed overlaps between genes upregulated by FOXA1 and the predicted target genes of miR-100-5p or miR-125b-5p. C, RT-PCR assays showed mRNA levels of potential target genes in HK1 cells upon loss of miR-100-5p or miR-125b-5p. D, RT-PCR assays showed mRNA levels of potential target genes HK1/FOXA1 cells upon transfection of miR-100-5p or miR-125b-5p mimics. E, western blot assays showed transfection of miR-100-5p or miR-125b-5p mimics downregulated RASGRP3 or FOXN3 protein levels in HK1/FOXA1 cells. F, sequence analysis showed the binding sites for miRNAs in the 3'-UTR of RASGRP3 or FOXN3. G, dual-luciferase reporter assays showed that miR-100-5p or miR-125b-5p targeted to RASGRP3 or FOXN3, respectively. **P < 0.01.

Target genes of miR-100-5p or miR-125b-5p were predicted by TargetScan 7.2. The function relevance of these target genes were categorized by KEGG pathway analysis (Figure 5A). The predicted target genes of miR-100-5p were involved in MAPK signaling pathway, oocyte meiosis, colorectal cancer, and other pathways in cancer. The predicted target genes of miR-125b-5p were concerned with MAPK signaling pathway, TGF-β signaling pathway, ErbB signaling pathway, insulin signaling pathway, adipocytokine signaling pathway, Fc epsilon RI signaling pathway and so on. We intersected the predicted target genes of these two miRNAs with the mRNAs upregulated by FOXA1 in HK1 cells. According to the mRNA microarray data, RASGRP3, one out of the 59 of predicted targets of miR-100-5p, was up-regulated in HK1/FOXA1 cells, whereas 6 out of 931 of predicted targets of miR-125b-5p (ABAT, C10orf54, FOXN3, KIAA1841, PAX9, and TLE3) were up-regulated in HK1/FOXA1 cells (Figure 5B). We demonstrated that inhibition of miR-100-5p led to increase of RASGRP3 mRNA level, and inhibition of miR-125b-5p led to upregulation of ABAT, C10orf54, FOXN3, KIAA1841, and TLE3 mRNA levels in HK1 cells (Figure 5C). Restoration of either miR-100-5p or miR-125b-5p in HK1/FOXA1 cells resulted in down-regulation of RASGRP3 and FOXN3, respectively (Figure 5D). However, transfection of miR-125b-5p mimics failed to downregulate ABAT, C10orf54, KIAA1841, and TLE3 mRNA levels in HK1/FOXA1 cells (Figure 5D). Furthermore, we provide evidence that FOXA1 upregulated both RASGRP3 and FOXN3 protein levels in HK1 cells, whereas miR-100-5p or miR-125b-5p mimics suppressed RASGRP3 or FOXN3 protein level in HK1/FOXA1 cells, respectively (Figure 5E). Therefore, RASGRP3 and FOXN3 were selected for further validation. Sequence analysis suggested that 292-298 nt at RASGRP3 3' UTR, 1584-1590 nt at FOXN3 3' UTR are predicted to be potential binding sites for miR-100-5p or miR-125b-5p, respectively (Figure 5F). Co-transfection of miR-100-5p or miR-125b-5p with luciferase reporter plasmids containing wild type of 3' UTR of either RASGRP3 or FOXN3 gene led to significant reduction in luciferase activity, whereas exerted no effect on luciferase activity of mutant 3' UTR lack of miRNA binding sites (Figure 5G). Furthermore, immunohistochemistry assays showed that RASGRP3 or FOXN3 protein level was increased in xenograft tumor tissues derived from either miR-100-5p or miR-125b-5p silenced HK1 cells (Figure S3).Thus, our data confirmed that RASGRP3 or FOXN3 was bona fide target of miR-100-5p or miR-125b-5p, respectively.

### Upregulation of RASGRP3 and FOXN3 contribute to FOXA1 mediated NPC cells suppression

**Figure 6:**
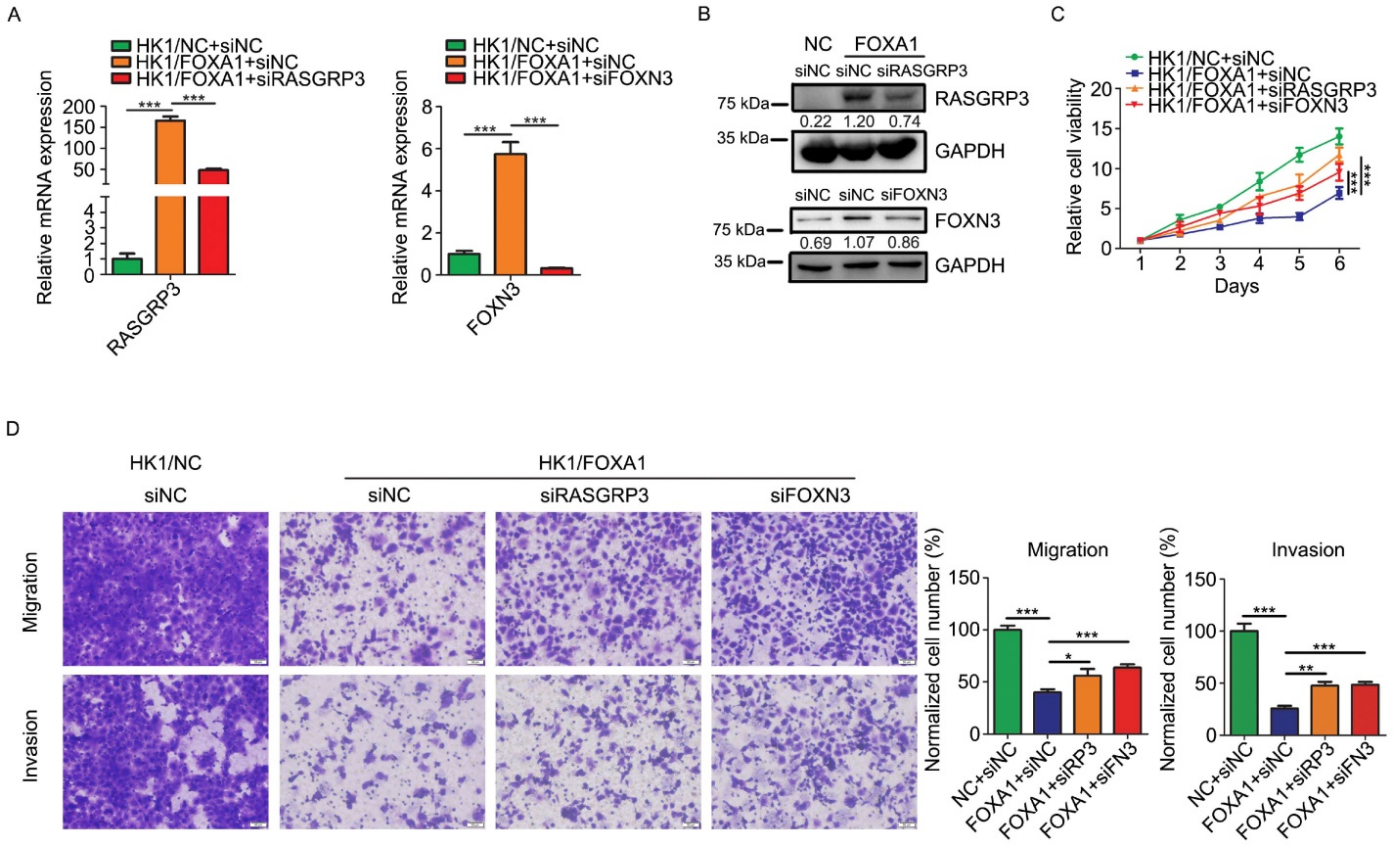
** Loss of RASGRP3 or FOXN3 restored cell proliferation, migration and invasion in HK1/FOXA1 cells.** A, RT-PCR assays showed transfection of specific siRNAs reduced mRNA levels of RASGRP3 or FOXN3 in HK1/FOXA1 cells. B, western blot assays showed transfection of specific siRNAs reduced protein levels of RASGRP3 or FOXN3 in HK1/FOXA1 cells. C, CCK-8 assay showed that silencing RASGRP3 or FOXN3 restored cell viability in HK1/FOXA1 cells. D, Transwell assays showed that loss of RASGRP3 or FOXN3 enhanced migration or invasion in HK1/FOXA1 cells. *P < 0.05, **P < 0.01, ***P < 0.001.

We then asked whether upregulation of RASGRP3 or FOXN3 contribute to the tumor suppressive role of FOXA1 in NPC cells. We demonstrated that siRNAs targeting to either RASGRP3 or FOXN3 inhibited its mRNA levels and protein levels in HK1/FOXA1 cells (Figure 6A & B). Functionally, silencing either RASGRP3 or FOXN3 in HK1/FOXA1 cells led to restoration of cell growth (Figure 6C). Furthermore, forced expression of FOXA1 inhibited migration and invasiveness in HK1 cells, whereas silencing RASGRP3 or FOXN3 in HK1/FOXA1 cells resulted in recovery of cell migration and invasion (Figure 6D). Hence, upregulation of RASGRP3 and FOXN3 at least partially contribute to FOXA1 mediated suppression of NPC cells.

**Figure 7:**
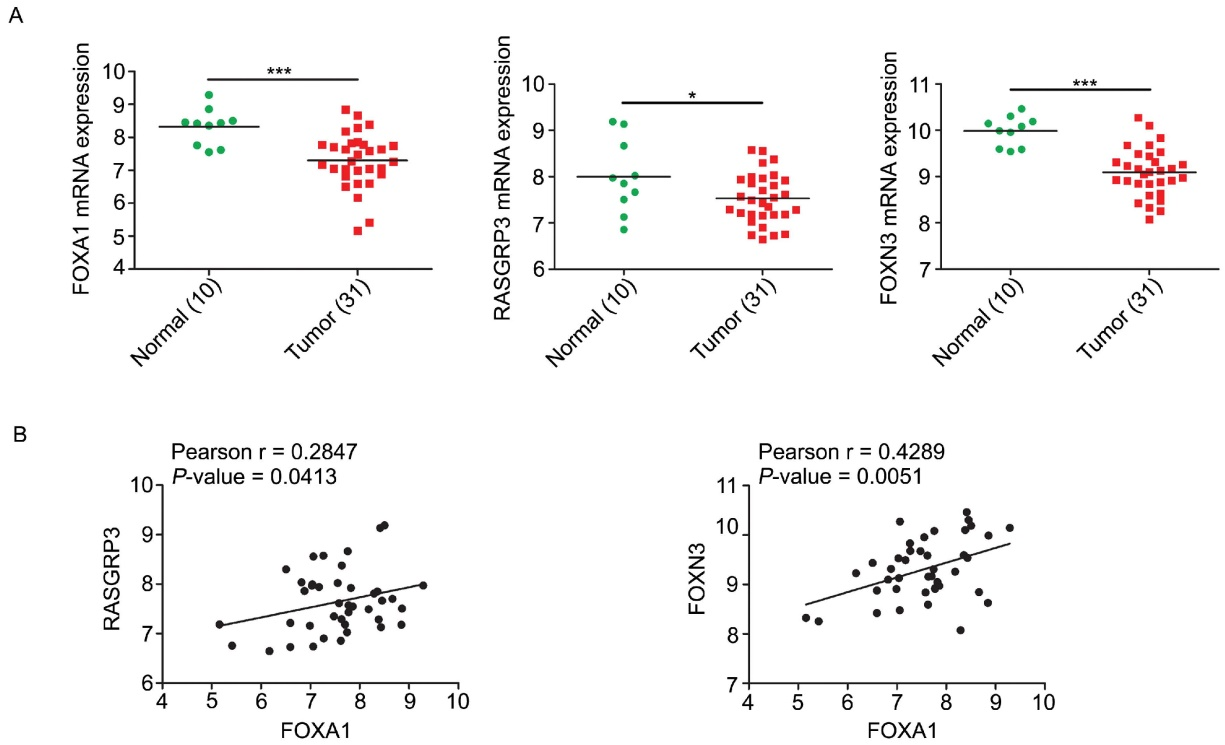
** Correlations of FOXA1, RASGRP3 and FOXN3 mRNA levels in NPC.** A, the mRNA levels of FOXA1, RASGRP3 and FOXN3 were down-regulated in NPC samples. Data were collected from GEO database (GSE12452). B, the mRNA levels of RASGRP3 and FOXN3 were positively correlated with FOXA1. *P < 0.05, ***P < 0.001.

We analyzed the expression levels of FOXA1, RASGRP3, and FOXN3 mRNAs in GEO database from NPC tissues. In accordance with expectation but perfectly logically and reasonably, the data showed that FOXA1, RASGRP3 and FOXN3 all have elevated expression in normal tissues and decreased expression in NPC tissues (Figure 7A). Correlation analysis displayed that both RASGRP3 and FOXN3 was significantly positively correlated with FOXA1 (Figure 7B).

## Discussion

Roles of FOXA1 in NPC development are rarely studied. In this study, we demonstrated that restoration of its expression in NPC cell HK1 lack of endogenous FOXA1 expression induced remarkable transcriptomic changes, including miRNAs and mRNAs. FOXA1 repressed two oncogenic miRNAs, miR-100-5p and miR-125b-5p, to upregulate their corresponding target genes (RASGRP3 or FOXN3, respectively) expression in HK1 cells. Functionally, repression of miR-100-5p and miR-125b-5p or activation of RASGRP3 and FOXN3 contributed to inhibition of malignant behaviors of NPC cells by FOXA1.

In consideration of the possibility that FOXA1 may directly activate RASGRP3 and FOXN3 transcription, we analyzed the ChIP-seq of FOXA1 performed in HepG2 cell (GSE91618) and found that FOXA1 does not bind to the genomic sequences of RASGRP3 and FOXN3, suggesting that FOXA1 does not directly regulate the transcription of RASGRP3 and FOXN3 (Figure S4). However, ChIP-seq data clearly indicated that FOXA1 directly binds to the upstream regulatory regions of miR-100-5p and miR-125b-5p, suggesting that FOXA1 may repress the transcription of miR-100-5p and miR-125b-5p through directly binding to the promoter regions of miR-100-5p or miR-125b-5p (Figure S4).

MiR-100-5p and miR-125b-5p are well-known oncogenic miRNAs in various human cancers. For example, miR-100-5p is significantly upregulated in renal cell carcinoma (RCC) tissues and exerts an oncogenic role in RCC 18. High expression of miR-100-5p in EML4-ALK positive non-small cell lung cancer (NSCLC) cells confers resistance to ALK tyrosine kinase inhibitors. Blocking miR-100-5p significantly augments the anti-tumor activity of ALK tyrosine kinase inhibitors to EML4-ALK positive NSCLC 19. High expression of mir-100-5p predicts unfavorable clinical outcome in oral squamous cell carcinoma patients 20. In this study, we provide evidence that stable inhibition of miR-100-5p suppressed cell viability, migration, invasiveness of HK1 cells in vitro and xenograft tumor formation in vivo, indicating an oncogenic role in NPC. Thus our study further supports its contribution to diverse cancer development and progression. The roles of miR-125b-5p are controversial. It has been shown that miR-125b-5p exerts tumor suppressive role in laryngeal squamous cell carcinoma through repressing hexokinase-2 and glycolysis 21. However, overexpression of miR-125b-5p in human cutaneous T-cell lymphoma (CTCL) remarkably enhances xenograft tumor growth and shortens survival of tumor bearing animals 22, suggesting an oncogenic function in CTCL. In this study, we found silencing endogenous miR-125b-5p attenuated the malignant behaviors of NPC HK1 cells. The discrepancy of miR-125b-5p functions in diverse cancers might reflect the remarkable genetic heterogeneity between different cancer types. On the other hand, miRNA's function is highly dependent on its target genes. One miRNA could target to hundreds of mRNAs, which may have distinct functions and various tissue expression patterns. Thus, it's reasonable that miRNA's function is cell-context dependent. We provide experimental evidence that restoration of either miR-100-5p or miR-125b-5p impaired the tumor suppressive activity of FOXA1 in HK1 cells. Thus, our study provides a new perspective to understand the mechanism of FOXA1 in tumorigenesis. Further work need to decipher the mechanisms underlying the regulation of miR-100-5p or miR-125b-5p by FOXA1.

We demonstrated that RASGRP3 and FOXN3 were identified as direct target genes of miR-100-5p or miR-125b-5p, respectively. RASGRP3 is an activator of Ras and is overexpressed in several human cancers 18, 23. We found that RASGRP3 mRNA level is moderately reduced in NPC samples as compared to its normal counterpart, which suggested that its function in NPC might be distinct from that in other tumors. We also found that forced expression of FOXA1 upregulated RASGRP3 at least partially through repressing miR-100-5p. Transient depletion of RASGRP3 in HK1/FOXA1 cells restored cell viability, migration and invasiveness. To date, we could not clearly explain the discrepancy of RASGRP3 function in NPC and other tumors. It has been shown that FOXA1 is upregulated in both replicative and oncogene-induced senescent cells 18. Forced expression of FOXA1 in endometrial cancer cells leads to cellular senescence 24. Sustained activation of Ras is known to be cause of oncogene-induced senescence 25, 26. Thus, we surmised that upregulation of RASGRP3 by FOXA1 in NPC is probably linked to cellular senescence mediated by FOXA1. However, this supposition needs to be validated by future experiments. FOXN3 belongs to forkhead box (FOX) protein family. FOXN3 acts as a tumor suppressor in a variety of human malignancies, including melanoma, breast cancer and hepatocellular carcinoma 27. In consistent with observations in other cancers, FOXN3 mRNA level is decreased in NPC samples. We identified that FOXN3 is a novel target of miR-125b-5p and FOXA1 upregulates FOXN3 in NPC cells through repressing miR-125b-5p. This data suggests that FOXA1 probably cooperate with FOXN3 to prevent NPC development.

## Conclusion

Our study uncovered an unrecognized role of FOXA1 regulated miRNA-mRNA networks in NPC biology, which shed light on the mechanisms underlying NPC development and provide potential therapeutic targets for NPC.

## Supplementary Material

Supplementary figures.
