# Supplementary material for: FOXA1 Suppresses the Growth, Migration, and Invasion of Nasopharyngeal Carcinoma Cells through Repressing miR-100-5p and miR-125b-5p
Source: J Cancer. 2020 Feb 10;11(9):2485–95. doi: 10.7150/jca.40709 (PMC7066028; doi:10.7150/jca.40709)
Supplement: Supplementary file 1 — Supplementary figures. [file jcav11p2485s1.pdf]

Supplementary materials

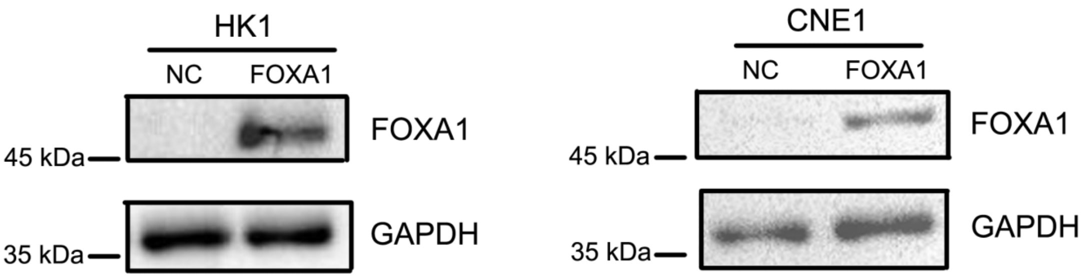

Figure S1. Protein level of FOXA1 in HK1 and CNE1 cells overexpressing FOXA1.

Western blot assays showed that FOXA1 protein levels were overexpressed in HK1/FOXA1 and CNE1/FOXA1 cells.

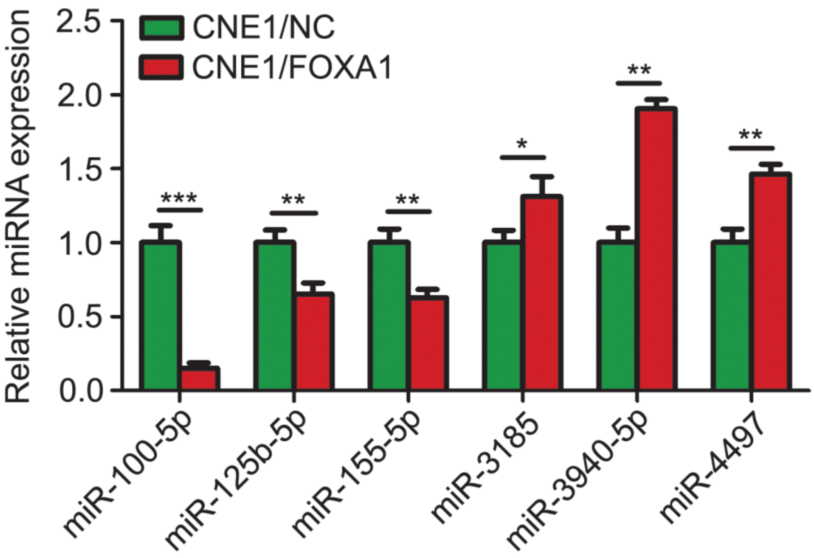

Figure S2. miR-100-5p and miR-125b-5p were down-regulated in CNE1 cells overexpressing FOXA1.

RT-PCR assay showed the validation of differentially expressed miRNAs in control or CNE1/FOXA1 cells. $*P < 0.05$, $**P < 0.01$, $***P < 0.001$.

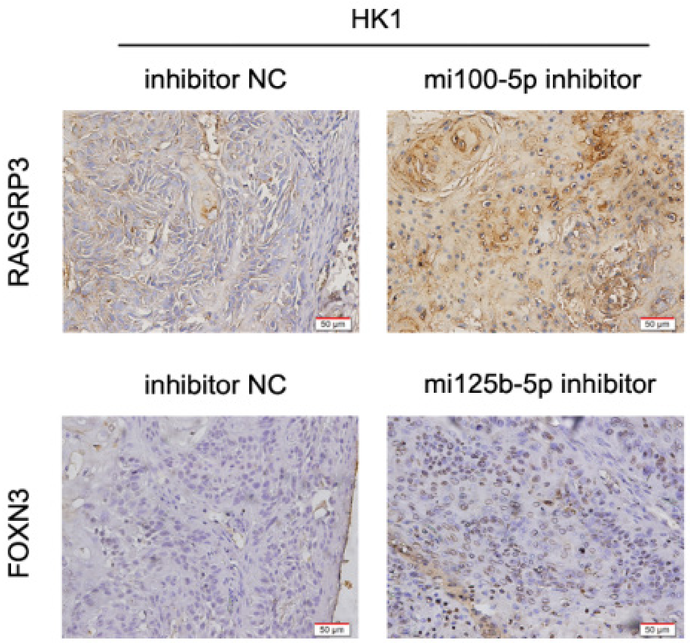

Figure S3. Protein level of RASGRP3 or FOXN3 was up-regulated in HK1 cells lacking either miR-100-5p or miR-125b-5p in vivo.

A, Immunohistochemistry assay showed that RASGRP3 or FOXN3 protein level was increased in HK1 cells knocking down miR-100-5p or miR-125b-5p in vivo.

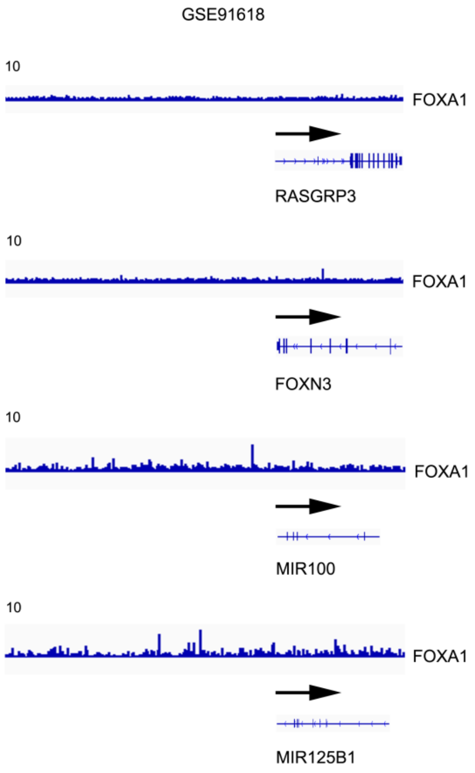

Figure S4. FOXA1 directly targeted to miR-100-5p or miR-125b-5p, not RASGRP3 or FOXN3.

A, ChIP-seq showed that FOXA1 didn’t combine with RASGRP3 or FOXN3, but it bound to promoter regions of miR-100-5p or miR-125b-5p. Data were collected from GEO database (GSE91618).
